# The Effect of Eye Contact Is Contingent on Visual Awareness

**DOI:** 10.3389/fpsyg.2018.00093

**Published:** 2018-02-07

**Authors:** Shan Xu, Shen Zhang, Haiyan Geng

**Affiliations:** ^1^Faculty of Psychology, Beijing Normal University, Beijing, China; ^2^Department of Psychology, University of Wisconsin–Whitewater, Whitewater, WI, United States; ^3^Beijing Key Laboratory of Behavior and Mental Health, School of Psychological and Cognitive Sciences, Peking University, Beijing, China

**Keywords:** eye contact, joint attention, gaze processing, implicit social cognition, visual awareness

## Abstract

The present study explored how eye contact at different levels of visual awareness influences gaze-induced joint attention. We adopted a spatial-cueing paradigm, in which an averted gaze was used as an uninformative central cue for a joint-attention task. Prior to the onset of the averted-gaze cue, either supraliminal (Experiment 1) or subliminal (Experiment 2) eye contact was presented. The results revealed a larger subsequent gaze-cueing effect following supraliminal eye contact compared to a *no-contact* condition. In contrast, the gaze-cueing effect was smaller in the subliminal *eye-contact* condition than in the *no-contact* condition. These findings suggest that the facilitation effect of eye contact on coordinating social attention depends on visual awareness. Furthermore, subliminal eye contact might have an impact on subsequent social attention processes that differ from supraliminal eye contact. This study highlights the need to further investigate the role of eye contact in implicit social cognition.

## Introduction

As a powerful social stimulus, eye contact communicates emotions and social intentions, such as approach-oriented motivations and general interests in the observer (Macrae et al., 2002; Adams and Kleck, 2005; Adams et al., 2006; George and Conty, 2008). Eye contact has been shown to capture attention more readily compared to averted gazes (Senju and Hasegawa, 2005; Vuilleumier et al., 2005; Conty et al., 2006), evoke positive affective responses (Bindemann et al., 2008; Willis et al., 2011; Chen et al., 2017), facilitate face recognition (Vuilleumier et al., 2005), positively affect attractiveness evaluation (Ewing et al., 2010), bias face preference (Jones et al., 2006), trigger self-referential processes (Conty et al., 2016; Hietanen and Hietanen, 2017), and eventually enhance interpersonal synchronization (Patterson, 1982). In most above-mentioned and many other laboratory studies, researchers often operationalize eye contact by presenting a direct gaze from a central face image to an observer. Although eye contact often functions as a signal facilitating social interaction, it can be also perceived as a threatening signal (Ellsworth et al., 1972; Emery, 2000) or has negative connotations. For instance, angry faces were rated less approachable and trustworthy when displaying direct gazes compared to averted gazes (Willis et al., 2011), and angry faces with direct gazes were categorized quicker and their emotion status judged to be more intense than those with averted gazes (Graham and LaBar, 2007; Sander et al., 2007; Bindemann et al., 2008).

Given the importance of eye contact, it is not surprising that eye contact can be processed at a subliminal level to modulate basic cognitive processes. For instance, subliminally presented eye contact facilitates visual awareness compared to averted gaze in interocular suppression, and is more capable of capturing attention as well as attracting eye movement than averted gaze (Stein et al., 2011; Chen and Yeh, 2012; Madipakkam et al., 2015). However, there is less investigation regarding whether and how eye contact modulates social cognition when the eye contact was presented without visual awareness. Given the functional diversity of eye contact, an intriguing question is whether the role of a subliminally presented eye contact would be different from that of a supraliminal eye contact. There has been evidence on different functions of eye contact with and without awareness in social decision making (Luo et al., 2016), but it is not clear whether such a dissociation is specific to that task, or can also be observed in other aspects of social cognition.

We explore this question by focusing on a fundamental aspect of social cognition: gaze-induced joint attention. Gaze-induced joint attention refers to the phenomenon that people automatically follow the averted gaze of others to align their attention (Driver et al., 1999; Nuku and Bekkering, 2008; Xu et al., 2011). As an essential non-verbal channel of interpersonal coordination, gaze-induced joint attention has been shown to emerge as early as in the first 3 months in infancy (Charman et al., 2000), and occurs automatically even if the averted gazes are task-irrelevant (Driver et al., 1999; Friesen et al., 2004; Frischen et al., 2007; Chen et al., 2017) or subliminally presented (e.g., Sato et al., 2007; Xu et al., 2011; Bailey et al., 2014; Mitsuda and Masaki, 2017; but see Al-Janabi and Finkbeiner, 2012 for evidence of the involvment of volitional control). Further, gaze-induced joint attention has been demonstrated to be specifically linked to the social relevance of gaze. It shows features distinct from that of non-social central attentional cues (Friesen et al., 2004), and it is modulated by social context such as the perceived intentionality of the gazer (Wiese et al., 2012, 2014; Wykowska et al., 2014). Also, gaze-induced joint attention recruits brain areas putatively involved in “Theory of Mind” (George and Conty, 2008), such as superior temporal sulcus and intraparietal sulcus (Vuilleumier et al., 2002; Calder et al., 2007) in addition to brain regions typically involved in attention-based orientation.

In the present study, we are interested in how this specific type of social attention might be affected if eye contact was presented prior to the averted gaze cues, and whether awareness of eye contact plays a moderating role. Supraliminal eye contact has been shown facilitating gaze-induced joint attention. Eye contact between two individuals can subsequently generate quicker attention orienting of one person following the gaze direction of the other, compared to when there is no such eye contact (Bristow et al., 2007). Also, gaze-induced joint attention following eye contact is accompanied by increased neural activity in regions critical in understanding communicative intent, such as medial prefrontal cortex (Schilbach et al., 2010; Caruana et al., 2015). Enhanced gaze-following behavior following eye contact is even found among 4–6 month-old infants (Farroni et al., 2003; Senju and Csibra, 2008). Given these findings, one might wonder whether such a potential impact of direct gaze may unconsciously occur, as eye-contact effects are thought to be partially triggered by subcortical regions (Senju and Johnson, 2009), which is less dependent on visual awareness.

To answer this question, we adopted a gaze-cueing paradigm, in which an averted gaze was used as an uninformative cue for a joint-attention task. Prior to the onset of an averted-gaze cue, either supraliminal (Experiment 1) or subliminal (Experiment 2) eye contact may occur. Following the literature (e.g., Kampe et al., 2003; Conty et al., 2006; Hietanen et al., 2008; Stein et al., 2011), we operationalized eye contact as a central face gazing directly at the observer. Experiment 1 extended previous research about eye contact’s facilitation effect on joint attention in that it examined the effect of task-irrelevant eye contact on subsequent joint attention. When Bristow et al. (2007) reported a facilitation effect of eye contact, their task was to judge the congruency of subsequent gaze shift in relation to a target. This task may have encouraged the participants to purposefully process gaze direction. In contrast, the present study rendered both eye contact and averted gaze cue task-irrelevant and therefore uninformative to participants, so as to examine whether and how eye contact affects subsequent joint attention automatically, without being intentionally processed. We expected to find the gaze-cueing effect, i.e., a shorter reaction time on detecting the location of a target following the congruent compared to the incongruent gaze cues. Furthermore, this gaze-cueing effect would be enhanced by the preceding supraliminal eye contact, despite of its task irrelevancy. Experiment 2 investigated the effect of subliminal eye contact on gaze-induced joint attention by rendering eye contact subjectively invisible with the technique of interocular suppression. We hypothesized that joint attention can also be modulated by subliminal eye contact.

## Experiment 1: the Effect of Supraliminal Eye Contact

In Experiment 1, participants viewed face stimuli containing averted gaze cues that were task-irrelevant, and reported the location of subsequent target stimuli. Half of the trials presented a face with direct gaze, which established an eye contact with the participant (the *eye-contact* condition); no such eye contact was established in the other half of the trials (the *no-contact* condition). Then the face displayed averted gaze, and participants were asked to report as quickly and accurately as possible the appearance of a target and its location, either left or right to the face. The direction of the averted gaze was either toward (in the *congruent* trials) or away from (in the *incongruent* trials) the target. The gaze direction was therefore independent of the location of the target and uninformative for participants to complete the task. Thus Experiment 1 adopted a 2 (Prior Eye Contact: *Eye-contact* vs. *No-contact*) × 2 (Gaze Congruency: *Congruent* vs. *Incongruent*) within-subject factorial design.

### Participants

Twenty-two students at Peking University participated in Experiment 1 (15 females and 7 males; 18–25 years, *M* = 21.8 years). The sample size was decided *a priori* based on earlier studies examining the behavioral effects of supraliminal eye contact (e.g., Xu et al., 2011). All participants had normal or corrected-to-normal regular and stereo vision.

Written informed consent was obtained from each participant before Experiments 1 (and 2). The experiments were approved by the ethics review committee at the Peking University.

### Stimuli

FaceGen (Copyright© 2009, Singular Inversions Inc.)’s Eastern Asian average male face with neutral emotional expression gazing slightly downward was used as the baseline face. It was trimmed to be 3° × 3°, showing the region from the top of the head to the upper neck. All other face stimuli were the same as the baseline face, except for their respective gaze directions. In the *eye-contact* face, the face image gazed straight ahead to form eye contact with the participants. In the *no-contact* face, the gaze direction was further down than in the baseline face, changed by the same degree as in the *eye-contact* condition. Two other face images, one gazing left and one gazing right, were used as the left and right averted gaze cues pointing to the possible locations of the target in each trial. The target was a picture of a toy duck, subtending visual angles of 1.5° × 1.5°, presented 8° left or right to the center of face stimuli. A light gray outer frame (19° × 19°) surrounded the face stimuli and the target throughout each block.

The stimuli were presented by Matlab 2009 with Psychtoolbox 3 on a 19 inch Viewsonic Professional Series P97f+ monitor (1024 × 768, visual angle 44.4° × 36.1°) connected to a Windows XP computer, against a dark gray (40, 40, 40 RGB) background.

### Procedure

**FIGURE 1:**
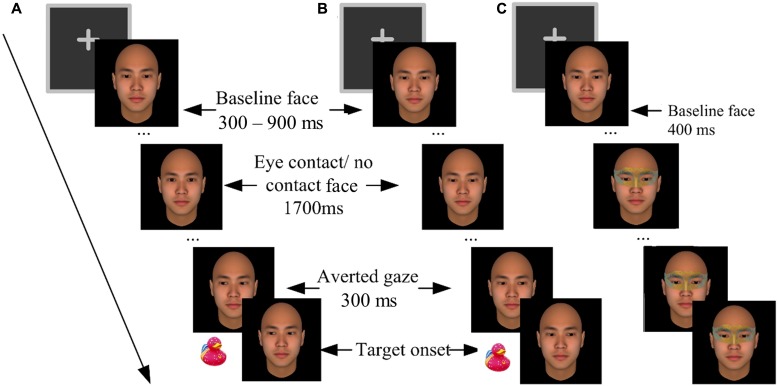
The procedures of Experiment 1. **(A)** An *eye-contact* + *congruent* trial (left-oriented gaze cue and left target). **(B)** A *no-contact* + *incongruent* trial (right-oriented gaze cue and left target). **(C)** A catch trial in which an eye mask was imposed onto the baseline face.

Participants completed the experiment individually with their heads supported by a chin rest at a viewing distance of 46 cm from the computer screen. They went through a practice block and five formal blocks. The blocks were separated by 1-min rest periods. The formal blocks contained 280 trials in total. Each formal block contained 28 critical trials, 7 catch trials, and 21 filler trials, all in a random order, in addition to four warm-up trials (not analyzed). Each critical trial (**Figures 1A,B**) started with a 400 ms presentation of a fixation cross. Then the baseline face appeared for 300–900 ms (randomly varied across trials), followed by two intermediate faces being presented for 100 ms each, and a 1700 ms presentation of the third face. In the *eye-contact* condition, the intermediate faces gazed gradually upward and the third face gazed straight ahead, forming eye contact with the participants. In the *no-contact* condition, however, the intermediate faces and the third face gazed further downward, making no eye contact with the participants. Then 300 ms before the onset of the target (a picture of a toy duck, 1.5° × 1.5° visual angles), another three faces with gradually averted gazes (left or right) were sequentially presented to create a perception of gaze shift. The target would randomly appear either left or right to the face.

Participants were instructed to indicate the location of the target (left or right) as quickly and accurately as possible, by pressing the “F” or “J” key with their left or right index finger, respectively. They were also informed in advance that the gaze direction was irrelevant to their task, and they should fixate on the central face, pay attention to bilateral areas on the screen, and avoid shifting their gaze during each trial. Both the target and the averted-gaze face image remained on the screen until a response was made or until 2000 ms had elapsed. Participants’ reaction time (RT) and accuracy of judgment were recorded. An incorrect response would prompt a warning message.

Filler trials were included to prevent participants from possible confounding effect on detecting the target, i.e., associating the onsets of the averted gaze and the target. One third of these trials presented an averted gaze cue but no target, and the rest presented a target but no gaze cue.

Catch trials were included to control for a potential confounding factor, i.e., the participants associating the onsets of the averted gaze with the target. These trials did not present any target or gaze cue; rather an eye mask would be imposed onto the baseline face 400 ms after its onset (**Figure 1C**). The participants were required to press the space key to report “masks,” to ensure that they were constantly paying attention to the eye region of the central face. To prevent guessing, the participants were warned beforehand that if their wrong location judgments or false alarms in the critical trials, or misses in the catch trials exceeded three times in a block, the block would terminate and restart^1^.

### Results and Discussion

**FIGURE 2:**
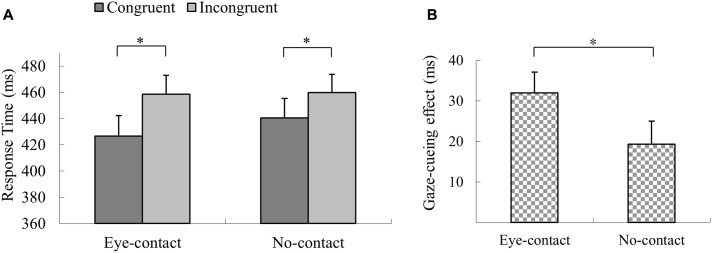
The results of Experiment 1. **(A)** Participants’ reaction times in Experiment 1. Reactions were quicker in the *congruent* than the *incongruent* trials in both the *eye-contact* and the *no-contact* conditions. **(B)** The gaze-cueing effect following supraliminal eye contact was larger than that in the *no-contact* condition in Experiment 1. The asterisks mark the statistically significant differences, *p* < 0.05.

The trials with incorrect response, with RTs shorter than 100 ms or outside three interquartiles’ range of each participant’s mean RT, were discarded (1.0% of all trials). A 2 × 2 repeated measures ANOVA was conducted with prior eye contact and gaze congruency as within-subject factors. The results revealed a significant main effect of gaze congruency, *F*(1,21) = 27.52, *p* < 0.001, $\eta_{p}^{2}$ = 0.57, with participants responding quicker in detecting the targets in the *congruent* condition than in the *incongruent* condition (*MD* = 25.7 ms), and a main effect of prior eye contact, *F*(1,21) = 5.34, *p* = 0.03, $\eta_{p}^{2}$ = 0.20, with the responses being quicker in the *eye-contact* condition than in the *no-contact* condition (*MD* = 7.6 ms). More importantly, the interaction between prior eye contact and gaze congruency was significant, *F*(1,21) = 7.81, *p* = 0.011, $\eta_{p}^{2}$ = 0.27. Simple effect analysis revealed that responses in the *eye-contact* trials were quicker in the *congruent* condition than in the *incongruent* condition, *F*(1,21) = 38.80, *p* < 0.001, $\eta_{p}^{2}$ = 0.65, *MD* = 32.0 ms, and this gaze congruency effect was also observed when no eye contact was presented, *F*(1,21) = 11.83, *p* = 0.002, $\eta_{p}^{2}$ = 0.36, *MD* = 19.4 ms (**Figure 2A**). The paired sample *t*-test was conducted to directly compare the sizes of the gaze-cueing effect (RT_incongruent_ – RT_congruent_) in the *eye-contact* and the *no-contact* conditions. The gaze-cueing effect was significantly larger when preceded by the *eye-contact* than the *no-contact* faces, *t*(21) = 2.80, *p* = 0.01, Cohen’s *d*_z_ = 0.60 (**Figure 2B**).

The results of Experiment 1 showed that supraliminal eye contact enhanced the subsequent gaze-induced joint attention compared to the *no-contact* condition. The enhancement of the gaze-cueing effect is in line with previous literature examining supraliminal eye contact (Bristow et al., 2007; Senju and Csibra, 2008). In Experiment 2 we proceeded to test whether subliminal eye contact would produce the same effect on the subsequent gaze-cueing effect.

## Experiment 2: The Effect of Subliminal Eye Contact

Prior research has demonstrated existence of subliminal processing of eye contact (Stein et al., 2011; Chen and Yeh, 2012; Luo et al., 2016; Chen et al., 2017). However, it is not clear whether and how subliminal eye contact affects subsequent joint attention. Using an interocular suppression technique termed Continuous Flash Suppression (CFS, Tsuchiya and Koch, 2005), Experiment 2 presented the first endeavor to investigate in particular the effect of subliminal eye contact on gaze-induced joint attention. In CFS, awareness of a stimulus presented to one eye is suppressed by a stream of noise images presented to the other eye. Compared to other paradigms such as masking and binocular rivalry, a remarkable advantage of CFS is that it induces robust and prolonged suppression, thus allows the suppressed stimuli to remain invisible for up to several seconds.

Using the same 2 × 2 within-subject factorial design (Gaze Congruency: *congruent* vs. *incongruent*; Prior Eye Contact: *Eye-contact* vs. *No-contact*) as in Experiment 1, Experiment 2 employed the same spatial-cueing paradigm, in which the presence of eye contact varies prior to a non-informative averted gaze cue. Unlike Experiment 1, the *eye-contact* face and the *no-contact* face were suppressed from participants’ awareness. The dependent variable was still the reaction time of target detection.

### Participants

Forty-six undergraduate students participated in Experiment 2 as paid volunteers. Data from 2 participants were excluded because of incomplete data recording; 12 participants were excluded because they did not pass the awareness test (see section “Procedure”). Data analysis was conducted on a final sample of 32 participants (19 females and 13 males; 18–25 years, *M* = 21.4 years). All participants had normal or corrected-to-normal regular and stereo vision.

The sample size of Experiment 2 was based on *a priori* power analysis. The effect size of eye contact on the gaze-cueing effect (Experiment 1) was used as a crude estimation of the same effect in Experiment 2, and *a priori* power analysis estimated a required sample of 32 participants to reach a power level of 0.9. Data collection was stopped after the number of valid participants reached this target. Although there was no past research on how subliminally presented eye contact affects subsequent gaze-induced joint attention for reference, our sample size was similar to those in previous studies investigating the gaze-cueing effect induced by subliminal averted gaze cues (e.g., Bailey et al., 2014; Mitsuda and Masaki, 2017; but see Al-Janabi and Finkbeiner, 2012 for smaller samples).

### Stimuli

**FIGURE 3:**
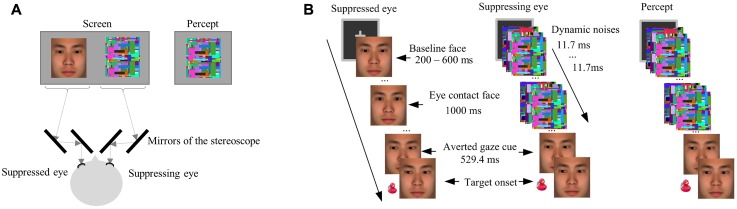
**(A)** A schematic drawing of the arrangement of the stereoscope and screen in Experiment 2. The stereoscope delivered different stimuli to each eye. The dashed line showed the path of light from the screen to the eyes of a participant. **(B)** An illustration of a critical trial in the *eye-contact* + *congruent* condition in Experiment 2. The target was presented left to the central face.

The face stimuli and target stimuli used in Experiment 2 were essentially the same as in Experiment 1. The only exception is that the faces used in the experimental session of Experiment 2 were enlarged (7° × 7°), including only the area from the forehead to the lower part of the jaw (**Figure 3**). This change in the face stimuli was to make the gaze stimuli stand out more so that any effect of eye gaze would likely be captured when the highly salient dynamic noise images were simultaneously presented during interocular suppression.

Noise images were generated for interocular suppression in sets prior to the experiment according to the following specifications: Each set of noise images started with a different Mondrian image containing randomly located squares of various sizes and colors (luminance: 30.8 cd/mm^2^), and then each subsequent noise image had more squares added to it, again of random colors and sizes and in random locations, to cover 10% of the image area. During the experiment, each set of images were presented in succession at the rate of monitor refreshing (85 Hz, i.e., each image lasted about 12 ms). Our earlier research has confirmed that such dynamic noise patterns generate robust interocular suppression for eye-gaze stimuli (Xu et al., 2011).

The interocular suppression was induced with the use of a stereoscope consisting of a set of mirrors, through which the stimuli were projected to the two eyes of the participant (see **Figure 3A** for the arrangement of the stereoscope). Specifically, the face stimuli were presented to the suppressed eye (the non-dominant eye of each participant) while the dynamic noise images of the same size presented to the corresponding location of the suppressing eye (the dominant eye of each participant, **Figure 3**), resulting in the interocular suppression (Tsuchiya and Koch, 2005; Jiang and He, 2006) that made the face stimuli (eye contact or not) invisible.

### Procedure

Upon arrival, participants were tested to determine their dominant eye, or randomly assigned one eye as the suppressed eye (vs. the suppressing eye) for those who did not show clear eye dominance. Each participant went through an experimental session flanked by two awareness test sessions, and always viewed the screen through a stereoscope. The viewing distance was the same as Experiment 1.

The experimental session was similar to Experiment 1. Five formal blocks contain 225 trials in total. Each formal block contained five warm-up trials, 36 critical trials, 3 catch trials, and 6 filler trials. The motivation of including the catch trials and the filler trials was the same as in Experiment 1, but the number of these trials were reduced to control the experiment duration and prevent fatigue, which may affect the strength of interocular suppression. In each critical trial, after a 400-ms binocularly presented fixation, the baseline face was presented for 200 – 600 ms (randomly varied across trials) to the suppressed eye of the participant, followed by either the *eye-contact* or *no-contact* face presented for 1000 ms, while dynamic noise images were flashed to the suppressing eye. Such a technique of interocular suppression made the face stimuli (the *eye-contact* face or the *no-contact* face) invisible (Tsuchiya and Koch, 2005; Jiang and He, 2006). Then, the face images with uninformative averted gaze cues were presented binocularly and thus visible to the participants, followed by the target after 529.4 ms. This presentation resulted in a perception that at the beginning of each trial, only dynamic noise images were presented, followed by the presentation of a face with left or right gaze (i.e., the averted gaze cue) and the target (**Figure 3B**). The contrast of the stimuli was adjusted individually to obtain reliable suppression effect, and the intermediate face images were omitted to simplify the stimuli presentation. The task was to indicate the location of the target (left or right) by pressing the corresponding keys as quickly and accurately as possible, the same as in Experiment 1. The participants were warned beforehand for the accuracy criterion, which was the same as in Experiment 1.

The awareness test sessions were to ensure that the participants included in data analysis experienced successful interocular suppression. Each awareness test consisted of 80 forced-choice trials, with half of the trials presenting the suppressed face images and the dynamic noise images in the same way as in the experimental session, and the other half replacing the suppressed face images with scrambled face images. The participants guessed at the end of each trial whether an intact face was presented, and if so whether it was an eye-contact face or not. Comparing a participant’s judgments in such a forced-choice task with the chance level has been recognized as a rigorous manipulation check for awareness (Merikle et al., 2001). If the responses deviated from the chance level in either awareness test session (chi-square tests, *p*s < 0.05), the participant’s data would be excluded from the final analysis.

### Results and Discussion

Thirty-two participants out of the 44 tested participants performed at chance level in the awareness tests. We reported the results of these 32 participants. Specifically, the mean accuracy of these participants on guessing whether there was an intact face presented in a trial was 50.7% (*SD* = 3.63), and the mean accuracy regarding the gazed direction was 53.40% (*SD* = 6.17)^2^. For the 32 valid participants, neither the sensitivity indices (*d*′) of discriminating between intact and scrambled faces (*Md*′ = -0.10, *SD* = 0.19) nor the *d*′ of discriminating between the eye-contact and the no-contact gazes (*Md*′ = -0.08, *SD* = 0.27) differed from 0 (binomial tests, *p*s > 0.10). These results demonstrated effective suppression of eye contact, therefore final analysis was conducted on the data in the experimental session from these participants.

**FIGURE 4:**
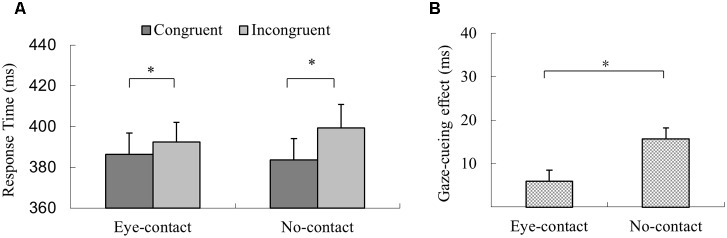
The results of Experiment 2. **(A)** Participants’ reaction times in Experiment 2. Reactions were quicker in the congruent than the incongruent trials in both the *eye-contact* condition and the *no-contact* condition. **(B)** Gaze-cueing effect following subliminal eye contact was smaller than that in the *no-contact* condition in Experiment 2. The asterisks mark the statistically significant differences, *p* < 0.05.

In the experimental session, trials with incorrect response, with RTs shorter than 100 ms or outside three interquartiles’ range of each participant’s mean RT, were discarded (2.9% of all trials). The 2 (Gaze Congruency: *congruent* vs. *incongruent*) × 2 (*Eye-contact* vs. *No-contact*) within-subject ANOVA revealed a significant main effect of gaze congruency with quicker responses in the *congruent* condition than in the *incongruent* condition (*MD* = 10.9 ms), *F*(1,31) = 27.42, *p* < 0.001, $\eta_{p}^{2}$ = 0.47. The main effect of prior eye contact was not significant, *F*(1,31) < 1, *p* = 0.39, $\eta_{p}^{2}$ = 0.02. Further, the interaction between prior eye contact and gaze congruency was significant, *F*(1,31) = 12.10, *p* = 0.002, $\eta_{p}^{2}$ = 0.28, but with a pattern different from that of Experiment 1. Simple effect analysis revealed that the responses in the *congruent* trials were quicker than the *incongruent* trials in the *no-contact* condition, *F*(1,31) = 39.30, *p* < 0.001, $\eta_{p}^{2}$ = 0.56, *MD* = 15.73 ms, as well as in the *eye-contact* condition, *F*(1,31) = 5.76, *p* = 0.023, $\eta_{p}^{2}$ = 0.16, *MD* = 6.00 ms (**Figure 4A**). To compare the sizes of the gaze-cueing effect in the *eye-contact* and the *no-contact* conditions directly, a paired sample *t*-test was conducted, which revealed that the gaze-cueing effect was significantly smaller in the *eye-contact* condition compared to that in the *no-contact* condition, *t*(31) = -3.48, *p* = 0.002, Cohen’s *d*_z_ = 0.61, different from the pattern observed in Experiment 1 (**Figure 4B**).

Experiment 2 revealed that subliminal eye contact *reduced* the subsequent gaze-induced joint attention compared to the *no-contact* condition, an impact opposite to that of supraliminal eye contact (Experiment 1). These distinct patterns may reflect a qualitative difference between the impacts of subliminal and supraliminal eye contact on subsequent joint attention. This result cannot be explained as a lack of processing of subliminal eye contact; otherwise there should not be a difference between the *eye-contact* and the *no-contact* conditions in Experiment 2. Second, it is unlikely that participants’ different response patterns between experiments were due to the difference in experimental setups, such as the presence of the dynamic noises in Experiment 2, since the dynamic noise images were similarly presented in the *no-contact* condition and the *eye-contact* condition. In addition, similar to Experiment 1, Experiment 2 revealed a significant gaze-cueing effect in the *no-contact* condition, suggesting sufficient processing of averted-gaze cues in this experiment. Another factor to consider is that Experiment 2 used a SOA different from Experiment 1 to accommodate interocular suppression. Both SOAs are within the range in which stable gaze-induced joint attention has been found (e.g., from 200 to 700 ms. Friesen and Kingstone, 1998; Driver et al., 1999; Frischen et al., 2007), and the longer SOAs may explain the shorter RTs in Experiment 2 compared to those in Experiment 1. Such reduction in average RTs in Experiment 2 may be the result of a standard foreperiod effect (Friesen and Kingstone, 1998; Bertelson, 1967). However, the change in average RTs cannot easily explain the even smaller gaze-cueing effect in the *eye-contact* condition compared to the *no-contact* condition in Experiment 2, because this factor is likely to affect both the *eye-contact* and the *no-contact* conditions, instead of producing a reversed effect of eye contact in Experiment 2.

## General Discussion

The present study reported dissociable effects of supraliminal and subliminal eye contact on gaze-induced joint attention: supraliminal eye contact increased subsequent gaze-cueing effect, but subliminal eye contact reduced it, compared with the corresponding *no-contact* conditions. The enhancement of the gaze-cueing effect following supraliminal eye contact is in line with previous literature (Bristow et al., 2007; Senju and Csibra, 2008), demonstrating the facilitation role of supraliminal eye contact in the alignment of social attention and interpersonal coordination (Patterson, 1982).

More importantly, Experiment 2 found reduced gaze-cueing effect following subliminally presented eye contact. Notably, the impact of subliminal eye contact was not a mere weakened version of that of supraliminal eye contact, neither did awareness simply amplify the effect of eye contact. Instead, the dissociation is in the directions of subliminal and supraliminal eye-contact effects. One speculation of ours is that subliminal eye contact may have social implications different from supraliminal eye contact. Existing research revealed that eye contact affects behavior in various ways, social facilitation being only one of it. For instance, eye contact can also be perceived as a threatening signal (Ellsworth et al., 1972; Emery, 2000). Subliminal eye contact evoked a larger negative potential at 200 – 300 ms in the partial-frontal area (Yokoyama et al., 2013), similar to subliminally presented fearful faces (Kiss and Eimer, 2008), suggesting that it probably conveys a negative rather than congenial social message. In line with this explanation, Luo et al. (2016) found that subliminally presented eye contact drove the individuals with a proself social value orientation to make less cooperative decisions compared to the *no-contact* condition, probably due to the ancient alarm reflex being evoked by the threatening implication of subliminal eye contact. Taking these findings, we speculated that the reduced gaze-cueing effect following subliminal eye contact might come from a mechanism similar to delayed attention disengagement induced by various threatening visual stimuli, including threatening words, emotionally threatening pictures, as well as supraliminal and subliminal fearful and angry faces (Fox et al., 2001; Yiend and Mathews, 2001; Carlson and Mujica-Parodi, 2015). In our study, subliminal eye contact might have functioned as a salient warning signal, thus similarly held attention and interfered with subsequent processes (such as the processing of averted gaze cues), and, consequently, reduced the following joint attention effect (the relative advantage of the *congruent* trials compared to the *incongruent* trials). If this speculation stands, our results would join existing evidence (Luo et al., 2016) in suggesting that the social meaning of eye contact may be contingent on visual awareness, and that the social-facilitation impact of eye contact may change remarkably in the absence of awareness. From this perspective, such a qualitative difference in the impact of eye contact at different levels of visual awareness may reflect the complexity of social cognition and the rich social implication of stimuli such as eye contact.

How can such awareness contingency of eye-contact effect exist? We speculated that visual awareness modulates the neural correlates of eye-contact processing. According to the fast-track modulator model (Senju and Johnson, 2009), eye contact is jointly processed by a subcortical pathway and a slow, deliberate cortical social brain network that is further subject to factors such as task demands and social contexts. Many brain regions in the cortical network are impacted by visual awareness. For instance, subliminal face stimuli elicited weaker neural activities in the prefrontal regions and the fusiform face area compared to supraliminal stimuli (Leopold and Logothetis, 1996; Tong et al., 1998; Jiang and He, 2006). In contrast, regions in the subcortical pathway have been demonstrated to respond to eye contact even in the absence of visual awareness (Madipakkam et al., 2015; Rothkirch et al., 2015). Thus, the effect of subliminal eye contact may mainly result from basic, reflective processing via the subcortical pathway, while the modulation from sociocultural norms may be weakened due to the limited access to certain cortical regions, as found in implicit social attitudes tasks (Greenwald et al., 1998; Nosek et al., 2002; Kiefer and Sekaquaptewa, 2007). On the other hand, the processing of supraliminal eye contact is subject to the modulation from top-down, context-specific inputs via the cortical network. As a result, different from the prosocial impact of supraliminal eye contact, a more reflexive warning function may manifest when the eye contact was subliminal, leading to a delay of attention disengagement or other types of fear response. In the present study this may lead to the reduction of subsequent gaze following response. This speculation can be further examined by directly testing the affective impact of subliminal eye contact or investigating the neural correlates of subliminal eye contact in the attention networks, the limbic system and other brain regions critically involved in affective processing.

The social account of the subliminal eye contact effect is but one potential explanation. The design of our study does not exclude alternative non-social explanations of our findings. For instance, in Experiment 1, it is possible that the gaze shift from the baseline face to the *no-contact* face might have distracted attention away from the central face, hence resulted in a smaller gaze cueing effect in this condition. Alternatively, the transitions from the baseline face to the *eye-contact* face or the *no-contact* face might be perceived as upward or downward biological motion, respectively, and the downward biological motion might be perceived as relatively subtle and may not facilitate the processing of subsequent averted gaze cue as much as the upward biological motion in the *eye-contact* condition. The subsequent gaze-cueing effect may have consequently reduced in the *no-contact* condition compared to the *eye-contact* condition. Further, in Experiment 2, the absence of visual awareness may compromise the perception of upward biological motion and affect the subsequent gaze-cueing effect. It may also be possible that the dissociations between upward and downward gaze shifts or biological motion are contingent on visual awareness or other aspects of the experimental setting. Exactly whether and how these factors may lead to the change of the direction of eye contact effect requires future research, for instance to investigate the neural activities in the attention networks and the cortical regions critically involved in gaze processing or the processing of biological motion while orthogonally manipulating the presence of gaze cues and visual awareness. Also, it is possible that inhibition of return (IOR) may have started to affect the gaze-cueing effect with the longer SOA in Experiment 2 (529.4 ms) than that in Experiment 1 (300 ms). Since IOR of centrally presented gaze cues was typically reported with much longer SOAs, (e.g., longer than 1 s, Frischen and Tipper, 2004; Frischen et al., 2007; McKee et al., 2007; Marotta et al., 2013; Hudson and Skarratt, 2016), future research is needed to explore whether IOR also affects responses as early as ∼500 ms, and further whether such an IOR effect can be moderated by eye contact and result in the reduced gaze-cueing effect compared to the *no-contact* condition.

Although there is also a study that failed to find evidence of the dissociation between subliminal and supraliminal eye contact (Chen et al., 2017), we speculate that it may be due to the specific research design with an affective priming task. The functional dissociation between supraliminal and subliminal eye contact may exist in certain, but not all, domains of social cognition. Taken together, research thus far indicates intricacy in the relation between visual awareness and social cognition that involves eye contact, and calls for future investigation to examine the functions of subliminal and supraliminal eye contact in specific contexts before generalizing findings from any single paradigm. Research in this direction might even shed light on the relation between awareness and sociality in general.

## Conclusion

Our study extends previous research investigating the potential dissociation between explicit and implicit social cognition into the research of eye-contact processing (Ewing et al., 2010; Luo et al., 2016). Utilizing one of the most basic, yet essential non-verbal form of social cognition, joint attention, we illustrated how the impact of eye contact may be contingent on visual awareness. We speculated that this contingency is because subliminal and supraliminal eye contact has different affective or social implications, a direction future research should continue to explore.

## Ethics Statement

This study was carried out in accordance with the recommendations of the Ethics Review Committee of Peking University with written informed consent from all subjects. All subjects gave written informed consent in accordance with the Declaration of Helsinki. The protocol was approved by the ethics review committee at the Peking University.

## Author Contributions

SX and HG proposed the ideas and designed the experiments. SX performed the experiments and analyzed the data. SX, SZ, and HG wrote the manuscript.

## Conflict of Interest Statement

The authors declare that the research was conducted in the absence of any commercial or financial relationships that could be construed as a potential conflict of interest.

## Footnotes

1 Two participants in Experiment 1 and four in Experiment 2 had to restart either the practice block or a formal block. No participant reported any difficulty in suppressing the gaze shift in the debriefing.

2 In contrast to the 32 valid participants, the participants who cannot pass the awareness tests performed well above the chance level with the mean accuracy in guessing intact faces were 86.37%, *SD* = 13.17, and the mean accuracy in guessing gaze direction was 63%, *SD* = 12.64.
